# A62 UNCOVERING MICROBIAL DETERMINANTS OF IBS FLARE-UPS: COHORT-LEVEL VS PATIENT-SPECIFIC APPROACHES

**DOI:** 10.1093/jcag/gwae059.062

**Published:** 2025-02-10

**Authors:** V Mohan, M pinto-sanchez, A Nardelli, R Borojevic, M Magee, M Shanmuganathan, Z Kroezen, G De Palma, P Moayyedi, P Britz Mckibbin, S Collins, P Bercik

**Affiliations:** McMaster University, Hamilton, ON, Canada; McMaster University, Hamilton, ON, Canada; McMaster University, Hamilton, ON, Canada; McMaster University, Hamilton, ON, Canada; McMaster University, Hamilton, ON, Canada; McMaster University, Hamilton, ON, Canada; McMaster University, Hamilton, ON, Canada; McMaster University, Hamilton, ON, Canada; McMaster University, Hamilton, ON, Canada; McMaster University, Hamilton, ON, Canada; McMaster University, Hamilton, ON, Canada; McMaster University, Hamilton, ON, Canada

## Abstract

**Background:**

Irritable bowel syndrome (IBS) is a complex gastrointestinal disorder with a global prevalence of 3.8% (5.8% in Canada, using Rome IV criteria), with the gut microbiota implicated in its pathophysiology. Despite extensive research in the last decade, the microbial mechanisms underlying symptom flares remain unclear. We showed in the previous analysis of our longitudinal study that the strongest factors responsible for separating IBS flare-ups from asymptomatic periods were stool appearance (Bristol stool scale) and somatization, followed by pain scores. Although there were no significant differences in alpha microbial diversity, beta diversity changes associated to symptom flares occurred in 25% of patients.

**Aims:**

Explore cohort-level versus personalized analyses in identifying microbial and metabolic predictors of flares in IBS patients.

**Methods:**

Twenty-eight IBS patients (IBS-D n=20; IBS-C n=8) and 10 healthy controls (HC) were followed for 25 weeks, with weekly symptom assessment (Birmingham IBS, HAD, STAI questionnaires) and stool sample collected (950 samples), analyzed by 16S rRNA sequencing and metabolomics profiling. Discriminatory taxa and metabolites among groups and within individuals were identified using sparse PLS-DA and mixed-effect models.

**Results:**

The integrated stool microbiome-metabolome analysis revealed 6 taxa and 8 metabolites in IBS-D, and 10 taxa and 18 metabolites in IBS-C cohort, that differed from HC (adjusted p<0.05). However, when comparing asymptomatic and flare-up timepoints within IBS cohorts, these microbial taxa and metabolites lost significance. Variance partitioning from mixed models indicated that individuals accounted for a larger share of the variance than cohort-level differences. Consequently, we performed time-course individual analysis in several patients, which identified about 40 bacterial taxa and 33 metabolites being altered during flare-up timepoints among both IBS-D and IBS-C patients. Furthermore, alterations in specific metabolic pathways were more prominent in individuals than in cohort comparisons.

**Conclusions:**

Our results suggest that personalized analysis is essential for better understanding of symptom dynamics within individual patients. Group-level differences may highlight general alterations in microbiome that make IBS patients more susceptible to the effects of transient taxa and metabolites identified in individual analyses, which might then contribute to symptom flares in that individual. While these microbial and metabolic predictors still require further investigation, they emphasize the complexity of IBS pathophysiology and the need for a hybrid approach to improve biomarker discovery.

**Funding Agencies:**

CIHR

